# Targeted Therapies and Immunotherapies in the Treatment of Esophageal Cancers

**DOI:** 10.3390/medsci7100100

**Published:** 2019-09-26

**Authors:** Adam Barsouk, Prashanth Rawla, Andreas V. Hadjinicolaou, John Sukumar Aluru, Alexander Barsouk

**Affiliations:** 1 Hillman Cancer Center, University of Pittsburgh, Pittsburgh, PA 15232, USA; adambarsouk@comcast.net; 2 Department of Medicine, Sovah Health, Martinsville, VA 24112, USA; 3 Academic Clinical Post-Doctoral Fellow and Gastroenterology Resident, MRC Cancer Unit and Department of Gastroenterology, University of Cambridge, Cambridge CB2 0XZ, UK; andreas.hadjinicolaou@gmail.com; 4 Senior Research Associate, Beth Israel Deaconess Medical Center, Harvard Medical School, Boston, MA 02212, USA; Jaluru@bidmc.harvard.edu; 5 Hematologist-Oncologist, Allegheny Health Network, Pittsburgh, PA 15212, USA; alexbarsouk@comcast.net

**Keywords:** esophageal cancer, metastatic, targeted therapy, immunotherapy, novel therapeutics

## Abstract

Esophageal cancer (EC) is among the most frequent and deadly cancers around the world. While esophageal adenocarcinoma (EAC) has one of the fastest-growing incidences amongst cancers in the US, it also has one of the lowest survival rates due to the limited effective treatment options. Fortunately, in the past decade, two targeted therapies and an immunotherapy agent have been approved by the FDA for metastatic EAC and esophageal squamous cell carcinoma (ESCC), with several more currently being considered for approval. In terms of immunotherapies, in July 2019, the FDA approved the PD1 inhibitor pembrolizumab for second-line treatment of PDL1-positive, advanced or metastatic ESCC. Two years before, pembrolizumab had been approved for the third-line treatment of PDL1-positive EAC. The PD1 inhibitor nivolumab, which was found in one study to outperform chemotherapy irrespective of PDL1 status, has yet to secure FDA approval. In terms of targeted therapies, although as many as 90% of EC cases show upregulated EGFR, anti-EGFR therapy has not been shown to improve survival. Ramucirumab, an antibody targeting both VEGF and HER2/neu receptors, has been approved for the treatment of refractory EAC, while the anti-HER2 monoclonal antibody (mAb) trastuzumab has been approved as front-line treatment for HER2-positive cases which account for approximately 20% of ECs. Although these targeted therapies and immunotherapies have resulted in significant improvements in survival for specific patient populations that are positive for certain biomarkers, such as PDL1 and HER2/neu, the survival rates remain low for a large proportion of the metastatic EC patient population, necessitating the development of further targeted treatment options.

## 1. Introduction

Esophageal cancer (EC) is among the deadliest neoplasms around the world. Although in the eighth position in terms of cancer incidence, EC ranks higher, at the sixth place, for cancer-related mortality [1]. The five-year survival rate in the US is only 20%, which is dismal when compared to a survival rate of 65% for colorectal cancer and 31% for gastric cancer. Unlike most gastrointestinal (GI) cancers (except for pancreatic cancer), EC treatment advances over the past 30 years have failed to have a clinical impact, resulting in less than double-digit improvements in survival [2]. Nevertheless, the future seems bright with three targeted therapies (including an immunotherapy agent) having been approved by the FDA for EC treatment in the last decade alone and several more drugs awaiting approval [3].

EC can be etiopathologically divided into esophageal squamous cell carcinoma (ESCC) and esophageal adenocarcinoma (EAC). ESCC affects the top two-thirds of the esophagus and is associated with risk factors such as alcohol consumption, drinking hot liquids, smoking, and achalasia. ESCC accounts for the majority of EC cases around the world and is mostly found in Asia and East Africa [4]. Cases of ESCC have been declining in many parts of the world with declining alcohol and tobacco consumption [1]. Incidence rises with age, and black individuals are 2–3 times more likely to develop ESCC compared to whites [5].

EAC, which affects the lower esophagus, is one of the cancers with the fastest rising incidence in the United States, increasing by as much as six-fold annually [2,4]. Adenocarcinomas of the gastro-esophageal junction (GEJ) are often grouped with EACs due to similar etiology. Both display tumorigenesis that seems to be initiated and promoted by gastroesophageal reflux, which in turn, is often associated with obesity. As such, EAC (including carcinoma of the GEJ) has now become far more incident than ESCC in the Western world and has been rising along with obesity rates, which have more than doubled among Americans over the past 40 years [4,6]. In addition to obesity and gastroesophageal reflux disease (GORD), EAC is also associated with Barrett′s esophagus, smoking, and achalasia [7]. Barrett′s esophagus is a pre-cancerous condition in which chronic injury due to persistent gastric reflux leads to intestinal cell metaplasia replacing the normal healthy squamous cell lining of the esophagus. Adenocarcinomas of the lower esophagus and GEJ arise from the simple columnar cells present in Barrett′s esophagus and thus share many features with gastric and intestinal adenocarcinomas. Consequently, targeted treatments proven effective for gastric and GEJ adenocarcinomas are widely considered effective for EAC [8].

According to a 2014 Cancer Genome Atlas study, EAC can be further divided into four etiological/genetic subtypes: 1. Epstein–Barr virus-associated tumors usually carrying phosphatidylinositol-4,5-bisphosphate 3-kinase catalytic subunit alpha (*PIK3CA*) mutations, 2. microsatellite instability (MSI) tumors commonly with *PIK3CA*, *EGFR* (epidermal growth factor receptor) and *HER2* (human epidermal growth factor receptor 2) mutations, 3. genomically stable gastro-esophageal cancers, characterized by *CDH1* and *RHOA* mutations and the *CLDN18–ARHGAP* fusion, and finally, 4. chromosomally unstable tumors with *TP53* mutations as well as receptor tyrosine kinase (*RTK)/RAS*, vascular endothelial growth factor receptors (*VEGFR*), and *p110* amplifications [9]. Many of the afflicted oncogenes and tumor suppressor genes have been revealed as promising targets for the development of targeted therapies and immunotherapies.

Of the 13 FDA-approved treatments for EC, 12 are associated with certain mutations and biomarkers, the presence of which significantly affects drug efficacy [3]. In contrast to most chemotherapies, that were only found to correlate with the aforementioned biomarkers post-factum, targeted therapies and immunotherapies are conceived and designed to bind specific molecules overexpressed by a patient’s tumor. Because of their widely differing and varying etiologies, EAC and ESCC have been considered unique and separate entities, and different targeted therapies have been investigated for the treatment of each one of them. Many such therapies, previously approved for different cancer types, appear to show promise for the notoriously lethal EC at present, offering hope of significant life extension in cases that were previously deemed non-treatable.

Chemotherapy for metastatic EC (mEC) is considered to be a palliative approach, meant to alleviate symptoms like dysphagia, aiming at improving symptom-free survival with limited if any at all, positive effect on overall survival. The most common cytotoxic approaches in the US involve combination therapies with a platinum-based agent like carboplatin, cisplatin, or oxaliplatin (which cross-link DNA and prevent replication), along with a taxel or with fluorouracil (5-FU) (which inhibit microtubule and nucleotide synthesis, respectively). The median overall survival (OS) for mEC in the US with cytotoxic and/or radiation therapy is only 8–10 months [10]. Consequently, there is an urgent, unmet need for targeted therapies in the treatment of mEC.

## 2. Targeted Therapies

### 2.1. EGFR Inhibition

EGFR is a tyrosine kinase receptor used in the signaling of cell growth, proliferation, migration, and metastasis. EGFR has been shown to be upregulated in 30–90% of ECs [11]. Recent studies have shown that 70% of EC have overexpressed EGFR [12,13]. In fact, in a 2004 study, EGFR-positive EC cases had a median OS of 16 months, less than half that of EGFR-negative cases (35 months), highlighting the importance of this molecule in disease progression and severity and supporting the biological rationale for urgently exploring EGFR-targeted therapies [14].

That same year, cetuximab, a chimeric monoclonal antibody (mAb) that binds EGFR extracellularly and inhibits ligand binding and cell proliferation signaling, was approved by the FDA for metastatic colorectal cancer (mCRC). Cetuximab, in combination with chemotherapy, has been shown to be effective at prolonging survival among patients with mCRC and head and neck cancers [10]. Unfortunately, cetuximab did not produce the same encouraging results for EC. A recent meta-analysis of 10 clinical trials over the past decade concluded that cetuximab (in combination with chemotherapy) did not demonstrate efficacy for improving survival of patients with either local or advanced EC, although it did improve the response rate in the latter [15]. Similarly, panitumumab, another anti-EGFR mAb, failed to improve survival in a phase III trial in which it was combined with chemotherapy [16]. In another phase III trial, gefitinib, an oral tyrosine kinase small-molecule inhibitor blocking EGFR, also failed to provide a survival benefit to EC patients [17]. Furthermore, nimotuzumab, another anti-EGFR mAb, although initially found to extend survival in combination with chemotherapy in a phase II trial, unfortunately failed to improve survival in a subsequent distinct phase III trial, leading to the trial′s early termination due to lack of a visible benefit [18]. The underwhelming results of EGFR targeted therapy have led to its loss of appeal as a potential and viable target for EC therapy.

### 2.2. VEGF Inhibition

Another promising targeted therapy that had previously been proven effective in the treatment of several GI cancers is the inhibition of vascular endothelial growth factor (VEGF). VEGF is the primary cytokine implicated in tumor angiogenesis, the process by which cancers stimulate the formation of new blood vessels to nourish the rapidly growing tumor as well as facilitate endothelial invasion and metastasis. Bevacizumab is a mAb targeting VEGFA, blocking it from binding VEGFR2 and stimulating angiogenesis [19]. Bevacizumab was approved for the treatment of mCRC in 2004 [20]. The addition of bevacizumab to cytotoxic agents (metabolite inhibitors and platinum-based chemotherapy) significantly improved survival and response rate for patients with mCRC [21,22,23]. Interestingly, among KRAS wild-type (WT) patients (the majority of CRC cases), EGFR inhibition (e.g., using cetuximab) was found to be more effective than VEGF inhibition through bevacizumab [24,25]. Given that as many as 99% of EC tumors are KRAS WT, it was originally thought that EGFR could be an ideal target in mEC [26]. However, 30–60% of mEC cases also display overexpression of VEGF, suggesting that VEGF targeting could also be effective in EC [27].

In 2011, the multinational AVAGAST study investigated bevacizumab in combination with chemotherapy in the first-line treatment of carcinomas of the GEJ, which is morphologically and pathologically similar to EAC. Although the trial concluded that bevacizumab did not significantly improve survival, it also revealed that while the drug was ineffective amongst Asian patients, it did improve survival amongst Pan-American patients, an effect that likely lies within the differences in the etiology of gastric and esophageal cancers between these populations [28]. This finding led to two subsequent phase III clinical trials, in which the proportion of enrolled Asian subjects was limited [29,30]. These confirmed that the VEGF/HER2 inhibitor ramucirumab, used as second-line monotherapy, could prolong OS for Western-world patients with gastric adenocarcinoma, including GEJ adenocarcinomas [31].

The HER2/neu receptor, a member of the same family of tyrosine kinase receptors as EGFR and targeted by ramucirumab in similar fashion to VEGF, was originally discovered to be overexpressed in about 30% of metastatic breast cancers and since then, it has been shown to be overexpressed in about 20% of EACs too [32,33]. On the basis of the aforementioned results with GEJ adenocarcinomas, ramucirumab has since been approved by the FDA for second-line treatment of EAC, either as monotherapy or in combination with the chemotherapy Abraxane [29,30,34]. Trastuzumab, an mAb targeting solely HER2, has also been approved for the frontline treatment of HER2-positive EAC patients in combination with chemotherapy [35].

Apitinib, a small-molecule inhibitor of both VEGFR and HER2, was recently evaluated in a Chinese study and found to significantly improve patient OS and progression-free survival (PFS) when compared to placebo in an Asian population with refractory GEJ adenocarcinoma [36]. An ongoing phase II trial for the front-line treatment of ESCC with apitinib, the PD1 inhibitor camrelizumab, and chemotherapy has reported promising results with a significant response rate to the combination, although patient survival data are immature and still being evaluated [37].

### 2.3. MET Inhibition

MET, the receptor for the stimulatory hepatocyte growth factor (HGF), has been shown to be associated with a worse prognosis for EAC when overexpressed in the tumor [38]. However, phase III trials of inhibitory anti-MET antibodies have so far failed to demonstrate any survival benefit [39,40].

## 3. Immunotherapy

Immunotherapies aim to enhance the body′s natural immune response by facilitating the targeting and destruction of cancer cells. Cytotoxic CD8 T-cells are able to recognize and eliminate cancer cells by inducing apoptosis or cell lysis. As cancer cells mutate, they evolve to evade the anti-tumor immune response (and, more specifically, the tumor-infiltrating CD8 T-cells) by developing immunosuppressive mechanisms that either inhibit, anergise, or trick cytotoxic T-cells into ignoring cancer and commit apoptosis themselves [41].

**Figure 1:**
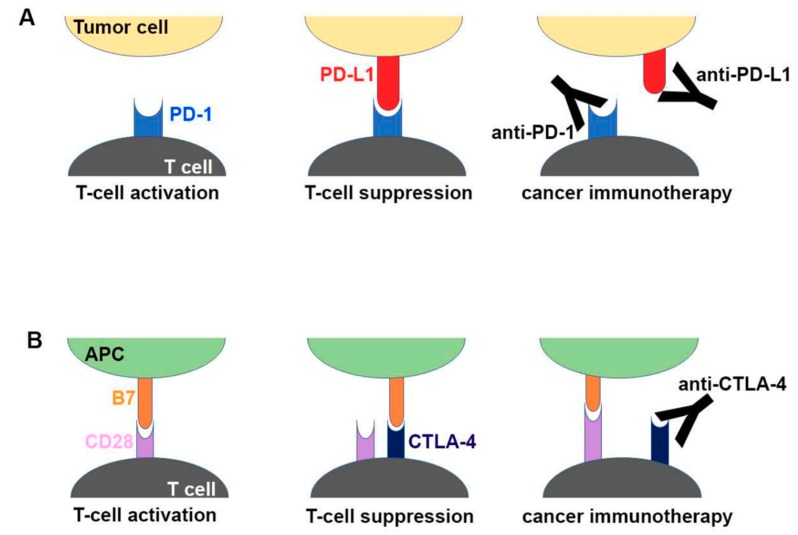
Schematic drawing of the molecular mechanism of checkpoint blockade by therapeutic antibodies for cancer immunotherapy. (**A**) T-cell activation is suppressed by the interaction between programmed death 1 (PD-1) on T cells and PD-L1 on tumor cells. Antibody drugs for cancer immunotherapy bind to PD-1 or PD-L1, blocking the PD-1–PD-L1 interaction. (**B**) T cells are activated by the interaction between B7 ligands of antigen-presenting cell (APC) and CD28 on T cells. Cytotoxic T lymphocyte-associated antigen 4 (CTLA-4) suppresses T-cell activation by competitive binding to B7. Therapeutic antibodies against CTLA-4 block the CTLA–4B7 interaction [46].

One of the suppressive immune evasion strategies employed by tumors to achieve this is by hijacking known natural immunosuppressive signaling pathways that are normally used as brakes or checkpoints to control immune cell function and prevent damage to the host during clearance of foreign microorganisms. One such checkpoint signaling molecule is programmed cell death protein 1 (PD-1), which acts as an inhibitory signaling receptor on the surface of T-cells. Cancer cells often overexpress the receptor′s ligand programmed cell death ligand 1 (PDL-1) in order to suppress lymphocyte activation and function and thus avoid T-cell-mediated destruction [42]. Targeting of PD-1 or PDL-1, known as checkpoint inhibition, has recently been shown to be an effective treatment for a variety of tumors including melanoma, lymphoma, lung cancers, head and neck cancers, and others that express significant levels of PDL-1 or induce PD-1 overexpression in T-cells [41]. In 2017, pembrolizumab, an mAb targeting PD-1, was approved for mCRC with high MSI and deficient in mismatch repair (MMR) [43]. Cancers with high MSI and deficient MMR have a high tumor mutational burden, leading to the development of neo-antigens that facilitate immune recognition. EC cancers often have a high tumor mutational burden, with almost half of the cases displaying p53 mutations (and many other genome abnormalities such as aneuploidy and cyclinA anomalies), likely due to the effects of chronic gastric reflux and inflammation driving continuous cellular and DNA turnover and, thus, eventually tumorigenesis [44]. Moreover, PDL-1 has been found to be expressed in around 40% of GEJ adenocarcinomas and is likely present in a similar proportion of EAC tumors [45]. For these reasons, EC has been considered a strong candidate for checkpoint inhibitor therapy. A schematic drawing of the molecular mechanism of checkpoint blockade by therapeutic antibodies for cancer immunotherapy is shown in Figure 1 [46].

Pembrolizumab, a PD-1 inhibitor, was initially assessed as first-line treatment in recurrent or metastatic gastric and GEJ PDL1-positive adenocarcinomas as part of a large, multicenter, phase Ib trial (Keynote-012) evaluating the drug in patients with various solid tumors [47]. Despite small numbers, with only 39 recruited patients, pembrolizumab showed promising anti-cancer activity with an objective response rate (ORR) of 22% (albeit all responses being partial) and OS of 11.4 months with a 12-month survival rate of 42%, as well as an acceptable safety profile with few severe adverse side effects [47].

The next checkpoint inhibitor evaluated was nivolumab, an mAb also targeting PD-1. Attraction-02, a randomized, double-blind, placebo-controlled, phase III trial involving patients from centers in Japan, Taiwan, and South Korea, evaluated nivolumab as third-line treatment in chemotherapy-refractory advanced, recurrent, or metastatic gastric and GEJ adenocarcinomas [48]. ORR for nivolumab was 11.2%, with an OS of 5.3 months compared to 4.1 months for placebo, suggesting that this immunotherapy agent offers a survival benefit at least within an Asian population and might be promising for patients with gastric and GEJ cancers where chemotherapy fails.

In Attraction-04 phase II trial, the same authors evaluated the role of combining nivolumab with chemotherapy in advanced or recurrent unresectable HER2-negative gastric or GEJ adenocarcinomas [49]. The combination of nivolumab with S-1 and oxaliplatin chemotherapy showed 57.1% ORR, with PFS of 9.7 months, whereas the combination of nivolumab with capecitabine and oxaliplatin had an ORR of 76.5% and a PFS of 10.6 months. Both combinations were tolerated well, and despite OS not being reached in either of the two tested groups, the response benefit suggested promising efficacy, and as such, the combinations are currently being tested in a phase III trial.

A study completed in January 2019 by the same group, furthered the case for checkpoint inhibition in EC, finding that nivolumab offered significantly higher OS and PFS relative to chemotherapy (docetaxel or paclitaxel) in a PDL1-unselected population with advanced or recurrent unresectable EC [50]. This was the first trial in EC to show a checkpoint inhibitor outperform chemotherapy in survival irrespective of the PDL1 status. As a result of these studies in an Asian population, nivolumab has been approved for PDL1-positive gastric and gastro-esophageal cancer patients in Japan and is expected to receive EC approval, whereas it has yet to secure FDA approval in the USA. 

The Checkmate 032 study assessed nivolumab in 160 patients with locally advanced or metastatic esophagogastric cancer (esophageal, gastric, and GEJ cancers) that had progressed under the standard of care (i.e., refractory to chemotherapy) [51]. The study found a 12% ORR and a 12-month survival rate of 39%, with median OS of around 7 months for nivolumab alone amongst all patients and intriguingly also revealed that the PD-L1 status did not significantly impact on the anti-tumor responses [51].

In addition to the assessment of nivolumab alone, the combination of nivolumab with ipilimumab, a distinct checkpoint inhibitor targeting the CTLA4 (cytotoxic T-lymphocyte-associated protein 4) receptor (another checkpoint molecule) was also evaluated. Previously, this combination had shown stronger responses but also a greater risk of immune-mediated adverse effects than nivolumab alone in the context of CRC [52]. In the same trial (Checkpoint-032), when compared to nivolumab alone, nivolumab plus ipilimumab combinations had greater progression-free survival (PFS; 10–17% vs 8%) and ORR (13–24% vs 12%) but similar OS, lower 12-month OS rates (24–35% vs 39%), and a significantly increased risk of adverse events (27–47% vs 17%) [53]. Although responses were observed irrespective of the PD-L1 status, the combination therapy also seemed to be significantly more beneficial in PDL-positive cases when compared to PDL1-negative cases, with ORR of 40% vs 22% for the nivolumab 1 mg/kg + ipilimumab 3 mg/kg cohort and 22% vs 0% for the nivolumab 3 mg/kg + ipilimumab 1 mg/kg cohort [53]. A similar trend was observed for the MSI status, with high-MSI tumors generally responding better than low-MSI ones. This is extremely interesting when considering that previous trials investigating the use of anti-CTLA4 as monotherapy in advanced gastric or GEJ adenocarcinomas had failed to show any benefit in comparison to best supportive care (BSC) [54]. This poor response was also observed in another phase II trial, this time using a different anti-CTLA4 mAb, tremenumab, which was tested as second-line treatment for metastatic gastric and esophageal adenocarcinoma [55]. In any case, as a result of the efficacy achieved when using anti-PD1 (nivolumab) and anti-CTLA4 (ipilimumab) checkpoint inhibitors together in phase I/II studies, the combination is currently being tested in a phase III trial [53].

Furthermore, Keynote-059, a non-randomized study evaluating the efficacy of pembrolizumab in patients with advanced (recurrent or metastatic), chemotherapy-refractory gastric and GEJ adenocarcinomas, displayed an ORR of 11.6% for all patients when the drug was used as third-line treatment [56]. ORR was 15.5% for PDL1-positive cases and only 6.4% for PDL1-negative cases. Interestingly, the combination of pembrolizumab with chemotherapy (cisplatin plus 5-FU or capecitabine) achieved an ORR of 60% for all patients, with PDL1-positive cases again having a significantly higher ORR compared to PDL1-negative cases (69% vs 38%). Finally, in the same trial, when pembrolizumab was used alone as first-line treatment in patients with PDL1-positive tumors, ORR was 26%, showing promising anti-tumor activity despite a higher rate of significant adverse side effects [57]. On the basis of the results of the Keynote-059 trial, the FDA approved pembrolizumab as third-line treatment of PDL1-positive, recurrent, metastatic, or locally advanced gastric and GEJ adenocarcinomas [58].

Unfortunately, a subsequent phase III randomized trial (Keynote-061) comparing pembrolizumab to third-line chemotherapy (paclitaxel) in patients with advanced gastric and GEJ cancer and a PDL1 combined positive score (CPS) of 1 or more, revealed that the former failed to significantly improve OS or PFS relative to third-line chemotherapy, despite having a better safety profile [59,60]. This finding suggested that the PDL1 status is not a good enough biomarker for base treatment decisions, and as such, pembrolizumab trials in gastric and GEJ adenocarcinoma are still ongoing.

More recently, in July 2019, the FDA approved pembrolizumab as a second-line option for PDL1-positive ESCC. The approval was based on the KEYNOTE-180 phase II and KEYNOTE 181 phase III trials. The KEYNOTE-180 trial found that pembrolizumab offered a significantly more durable response for metastatic ESCC (mESCC) patients who had undergone more than two lines of standard treatment, especially for those with PDL1 tumors [61,62]. The ORR was 14.3% for mESCC patients but only 5.2% for patients with metastatic esophageal adenocarcinoma. PD-L1 status was deemed important in this study, as patients with PDL1-positive tumors had a higher ORR than those with PDL1-negative tumors (13.8% vs 6.3%). A follow-up study, the global KEYNOTE-181 trial, found that pembrolizumab used in mESCC as second-line treatment, improved OS in an all patient analyses when compared to chemotherapy, although this effect was not statistically significant [63]. However, pembrolizumab was found to be superior to chemotherapy as second-line treatment in terms of OS when patients with PDL1 CPS > 10 were evaluated, highlighting the importance of PDL1 status [63]. These results have led to trials that will combine pembrolizumab and chemotherapy as first-line treatment of advanced EC.

Another immunotherapy studied in EC is avenumab, an anti-PDL1 mAb evaluated in the Javelin 300 phase III trial in a patient population with advanced (recurrent or metastatic) unresectable gastric or GEJ cancer that had not responded to two lines of chemotherapy [64]. Unfortunately, results were not encouraging, as the ORR for avenumab was worse than that for paclitaxel chemotherapy (4% vs 8%) when they were compared as third-line treatment; also, they had similar OS (4.6 vs 5 months).

Finally, a recent phase II trial found pembrolizumab in combination with trastuzumab, capecitabine, and oxaliplatin in the first line with significantly promising ORR (83%) and PFS (11.4 months) in HER2-positive metastatic EAC patients, paving the way for combinations comprising immunotherapy, targeted therapy, and chemotherapy for EC [62].

A recent meta-analysis confirmed that anti-PD1 and anti-PDL1 immunotherapies are significantly efficacious for patients with advanced, chemotherapy-refractory gastric or GEJ cancer and show even more promising anti-tumor activity and enhanced ORR when tumors overexpress PDL1 [65]. Similar to PDL1 overexpression, microsatellite instability, which can also highly predict the efficacy of checkpoint inhibitors in colorectal cancer, is also being evaluated for its predictive value in EC immunotherapies. Other immunotherapies, such as T-cell stimulators like OX40 and CD40 antibody agonists, are being investigated in translational and early-stage trials for EC. Approved targeted therapies and their associated biomarkers have been summarized in Table 1.

## 4. Conclusions

Esophageal cancer is among the deadliest neoplasms in the Western world and, despite new treatment options, it has not kept pace with other GI cancers in terms of improvements in survival. ESCC is far more common around the globe than EAC. However, EAC, which resembles gastric and GEJ adenocarcinoma, is more common in the Western world and rapidly increasing in incidence at alarming rates. Chemotherapy regimens for mEC are associated with survival of less than a year on average and are considered mainly palliative. The PD1 inhibitor pembrolizumab, VEGF + Her2/neu inhibitor ramucirumab, and HER2/neu inhibitor trastuzumab have been shown to improve survival or response rate in the treatment of advanced EC. Pembrolizumab has been demonstrated to be more effective than chemotherapy in late lines of treatment of both ESCC and EAC, leading to its approval as a second- and third-line treatment option for those etiologies, respectively. Ramucirumab has been approved as second-line treatment of EAC, while trastuzumab has been approved as first-line treatment in combination with chemotherapy for HER2/neu-positive patients. Survival for mEC remains low, and the development of further targeted therapies, especially for effective and durable first-line treatment, is urgently needed.

## Figures and Tables

**Table 1:** Approved targeted therapies and associated biomarkers in esophageal carcinoma.

Therapy	Trade Name	Cellular Target	Biomarker	Treatment
Pembrolizumab	Keytruda	PD-1	PDL1-positive mESCC	Second-line monotherapy
			PDL1-positive mEAC	Third-line monotherapy
Trastuzumab	Herceptin	HER2/neu	HER2-positive mEAC	First-line in combo w/chemotherapy
Ramucirumab	Cyramza	VEGF, HER2/neu	mEAC patients without cardiovascular comorbidities	Second-line, monotherapy or in combo w/Abraxane
